# Rare Repeat: A Case Report and Literature Review of Recurrent Intrathoracic Schwannomas

**DOI:** 10.7759/cureus.82511

**Published:** 2025-04-18

**Authors:** Daniel Hahn, Ginikachi Olelewe, Lauren Velasquez, Jasmine Fung, Margaret Lawless, Vinay Tak

**Affiliations:** 1 Medicine and Surgery, Touro College of Osteopathic Medicine, New York, USA; 2 Pathology, St. Mary General Hospital, Passaic, USA; 3 Cardiothoracic Surgery, St. Mary General Hospital, Passaic, USA

**Keywords:** intercostal nerve tumor, intrathoracic tumor, peripheral nerve sheath tumor, recurrent schwannoma, schwannoma, thoracic surgery

## Abstract

A schwannoma is a benign peripheral nerve tumor often located in the head and neck, posterior mediastinum, and, less frequently, in the chest wall. The recurrence of schwannoma at the same anatomical site after complete surgical resection is rare. This study discusses the case of a 60-year-old man with a recurring intercostal nerve schwannoma, which appeared eleven years after initial surgical resection in the same area of the chest wall. A second intrathoracic surgery was performed to remove a 6.0 x 3.0 x 2.5 cm partially encapsulated mass. The tumor was completely excised through a thoracotomy approach and revealed to be a schwannoma.

## Introduction

Schwannoma, previously known as a neurilemmoma, is a benign tumor that is encapsulated and originates from the peripheral nerve sheath, consisting of neoplastic Schwann cells. Schwannomas are benign tumors that are well-circumscribed, have smooth surfaces, and are attached to peripheral nerves [1,2]. Typically, these tumors appear as asymptomatic, slow-growing, and painless masses. Due to their slow growth and typical locations, schwannomas can be tolerated for an extended period until their size compresses adjacent structures [3]. While noninvasive, they have the potential to grow, causing varying degrees of dysfunction depending on the location. In these cases, more severe symptoms include superior vena cava syndrome and dysphagia [2].

Most schwannomas arise from cranial nerves and spinal nerve roots and often manifest in areas such as the head and neck, posterior mediastinum, and extremities. However, they can occasionally be found within the thoracic cavity, as seen in cases of chest wall schwannomas. Intrathoracic schwannomas often originate from sympathetic nerve trunks and intercostal nerves [4]. When located in the thorax, these tumors can mimic other thoracic masses, leading to delayed diagnoses until symptoms become significant or a biopsy confirms the diagnosis. Recurrence of a schwannoma in the same anatomical region is exceedingly rare, particularly following complete resection of the initial tumor [5]. 

Given the rarity of recurrence with intrathoracic schwannomas, this case underscores the crucial role of long-term follow-up in patient care. It presents a unique instance of two separate, recurrent intrathoracic nerve schwannomas occurring 11 years apart. This presentation highlights the importance of complete patient medical records and increased awareness of potential recurrence of atypical schwannomas.

## Case presentation

The patient, a 60-year-old man, presented with a right-sided anterior mass and a past medical history of hypertension and a right-sided schwannoma that was resected initially 11 years ago in his home country. He also had a 30-pack-year smoking history.

The initial presentation was 11 years ago, while the patient resided in his home country. An incidental right-sided mass was identified during a routine annual examination. At that time, he reported a right-sided tingling sensation in the subareolar region, occasional sharp pain, and infrequent burning sensations. The patient denied experiencing weight loss, changes in appetite, shortness of breath, or chest pain. A thoracotomy was carried out to resect the mass. The final pathology report indicated that the mass was a schwannoma.

**Figure 1:**
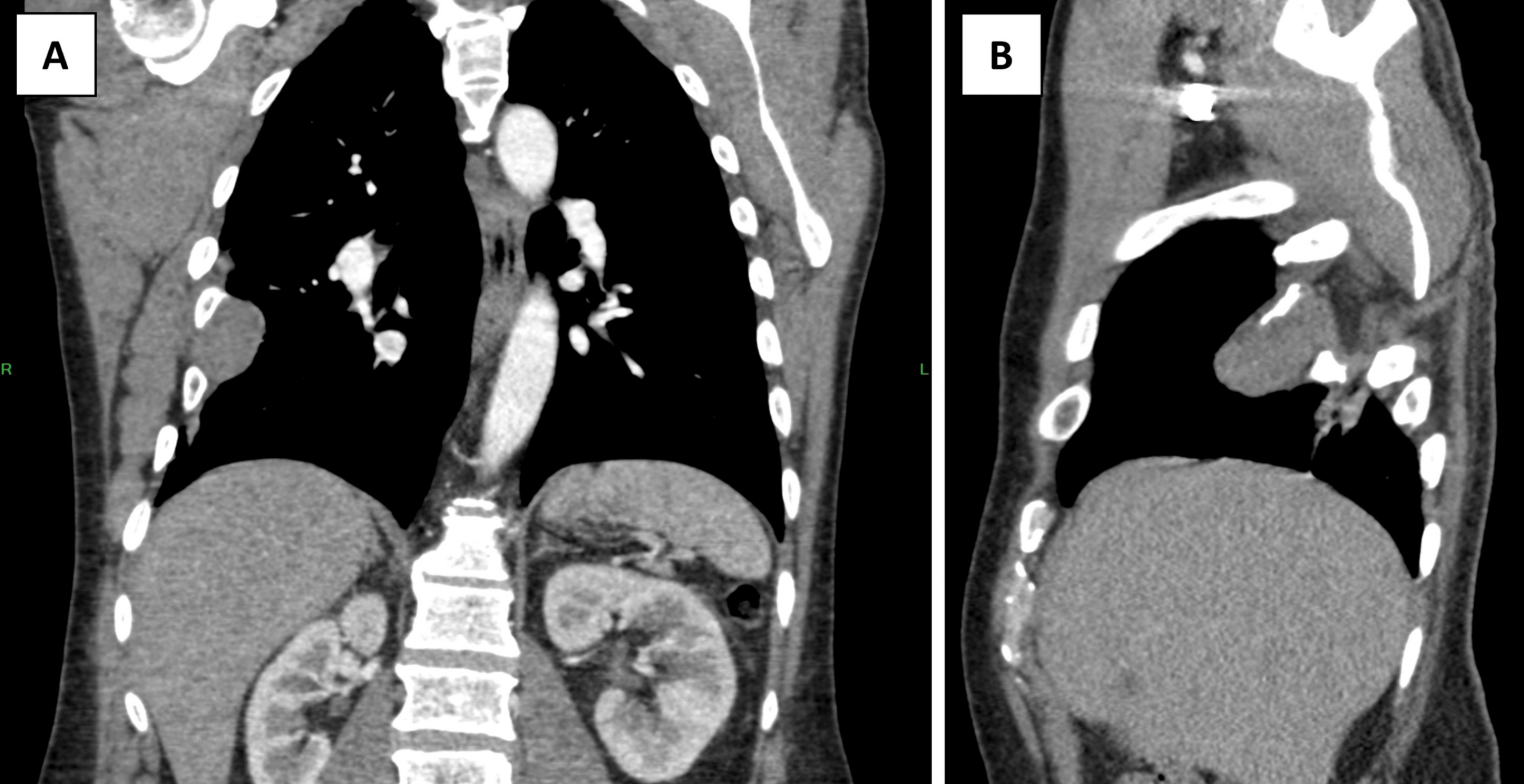
Pre-operative CT scan of the schwannoma to identify anatomical location CT: Computed tomography; A: Coronal plane; B: Sagittal plane

Eleven years later, in the present case, the patient presented with a complaint of elevated blood pressure, occasional right subareolar tingling, and a sensation of increased chest pressure. The patient denied weight loss, changes in appetite, shortness of breath, and chest pain. Because the patient had a past medical history of schwannoma in the same area, a computed tomography (CT) scan was ordered (Figures 1A, 1B), and it confirmed a right-sided mass in the seventh intercostal space.

**Figure 2:**
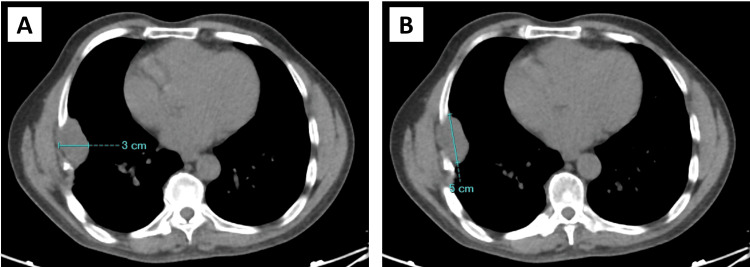
Measurement of the schwannoma based on low-dose CT scan CT: Computed tomography; pre-operative measurement using CT imaging estimated the size of the recurring schwannoma to be 3 cm in width (2A) and 5 cm in length (2B)

A low-dose CT (LD-CT) scan was also ordered, and the mass was initially measured to be 3 x 5 cm in width and length, respectively (Figures 2A, 2B).

**Figure 3:**
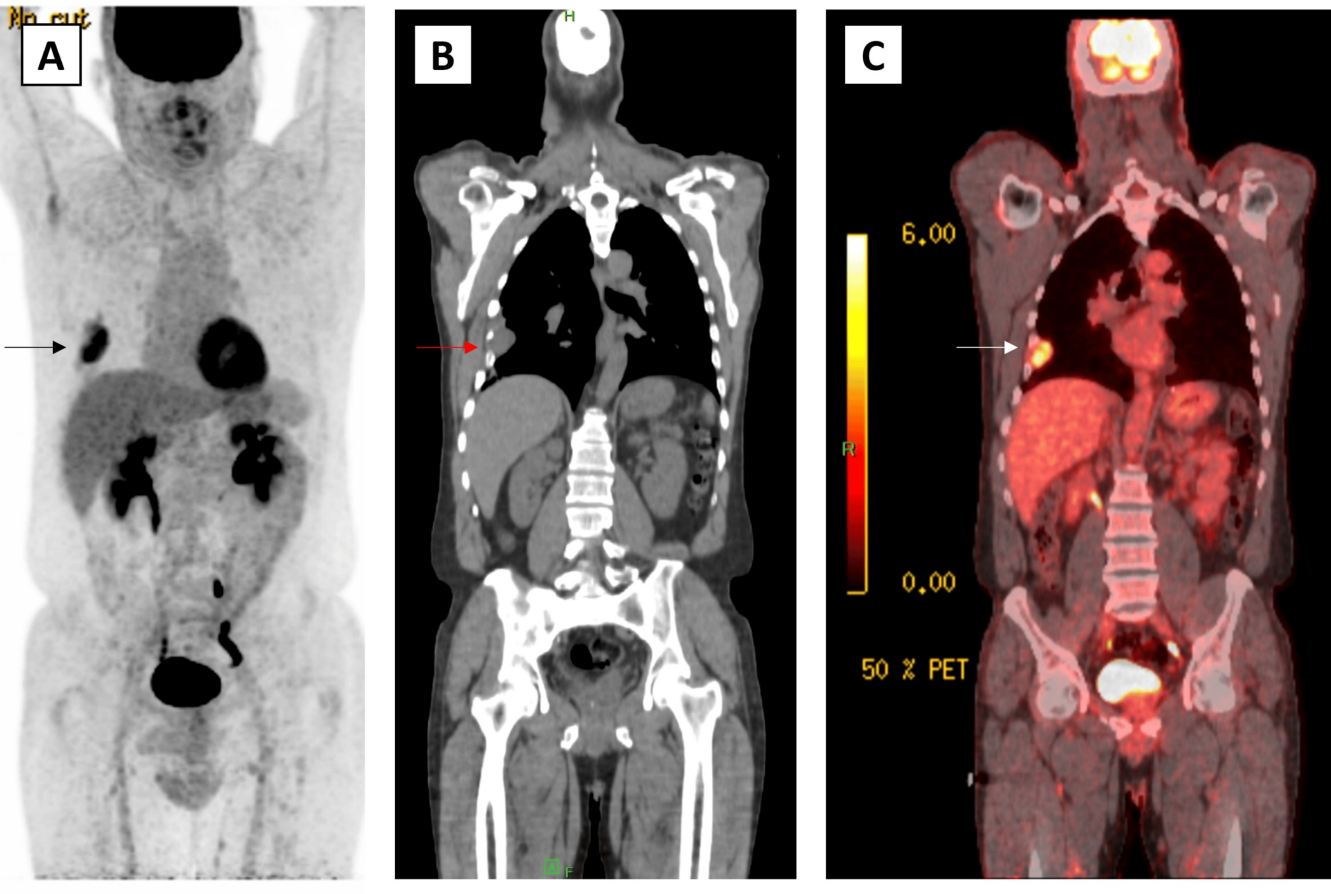
Preoperative MIP and PET/CT scans of the schwannoma to identify areas of potential metastatic spread and metabolic activity MIP: Maximum intensity projection, PET/CT: Positron emission tomography/Computed tomography; A: Coronal MIP scan, B: Coronal CT scan, C: fusion PET/CT scan, Arrows: schwannoma

Lastly, fusion positron emission tomography/computed tomography (PET/CT) and maximum intensity projection (MIP) scans were conducted to identify areas of potential metastasis and increased metabolic activity (Figures 3A-3C). Only the single mass in the seventh intercostal space was identified. With pre-operative imaging completed, the patient was scheduled for thoracic surgery.

Operation report

**Figure 4:**
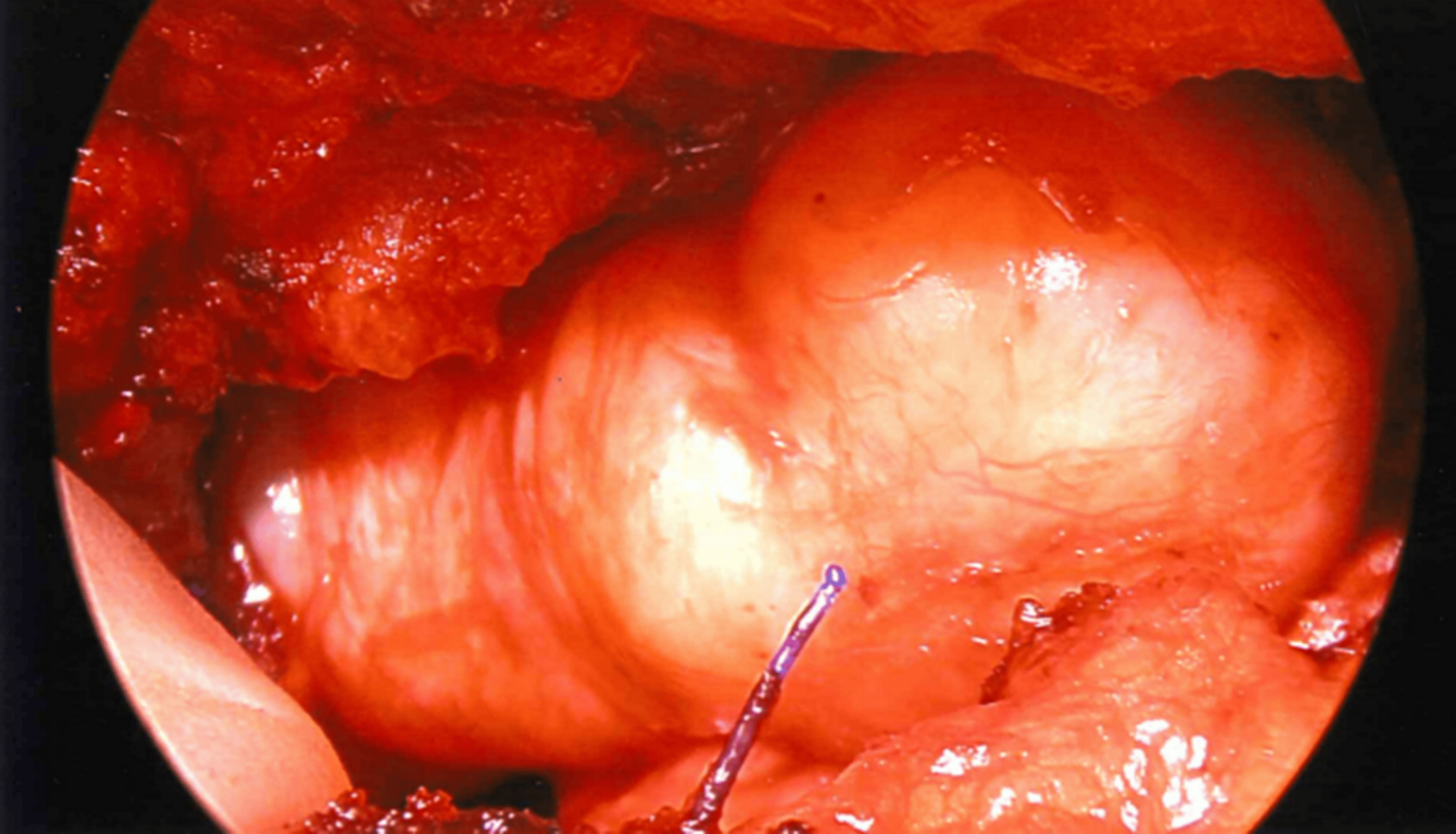
Appearance of the schwannoma via thoracoscopic view

The patient was positioned for a right-sided video-assisted thoracic surgery (VATS) procedure. A port site was placed in the seventh intercostal space in the mid-axillary line. Thoracoscopic visualization showed a mass in the same position as the preoperative PET-CT scan in the seventh intercostal space (Figure 4). 

**Figure 5:**
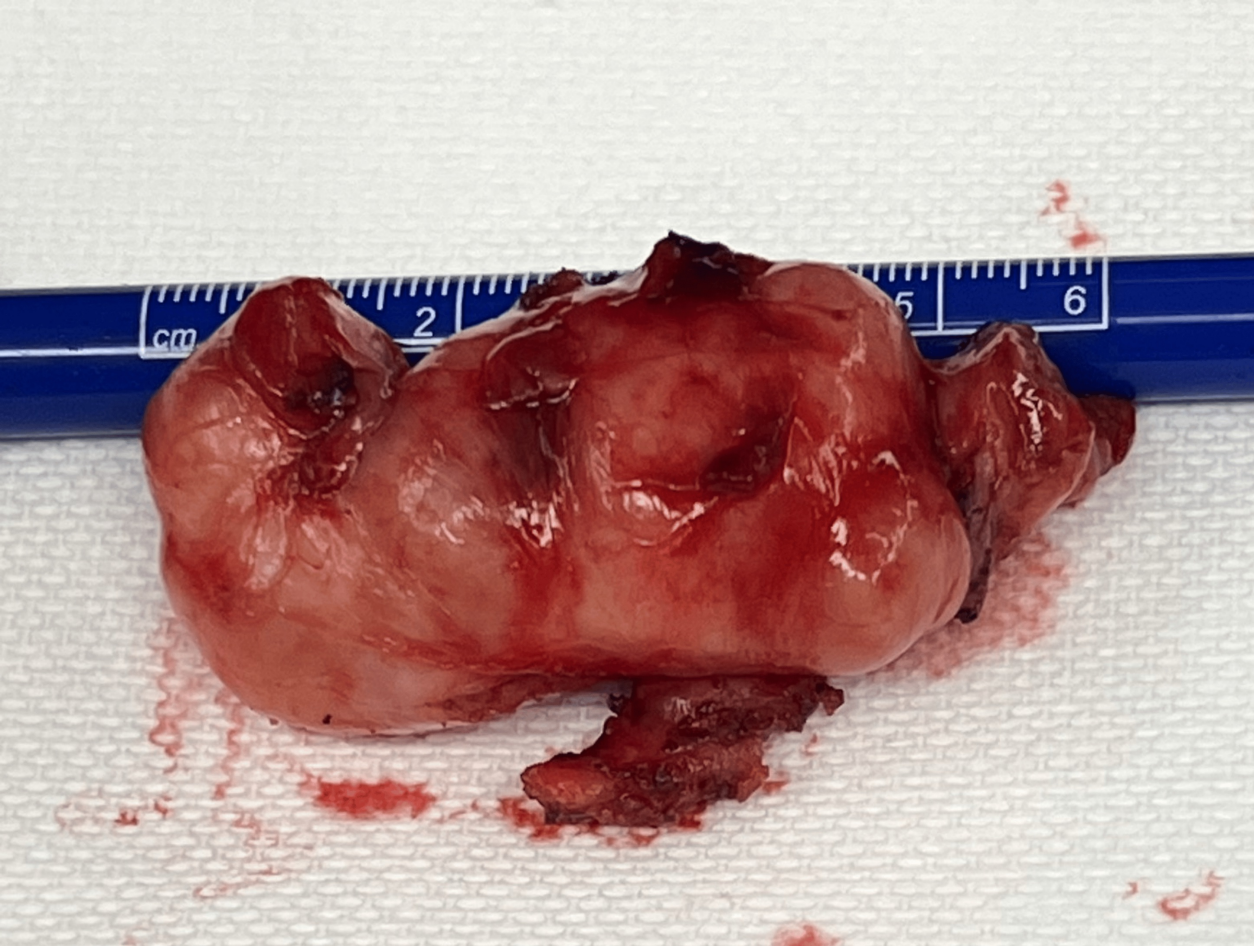
Gross appearance and measurement of the excised schwannoma

Due to the anterior location of the mass on the thoracic wall and modified anatomy from the previous surgery, a novel surgical approach was taken by separating the latissimus dorsi and serratus anterior muscles from one another. An additional entrance was created, two intercostal spaces superiorly, and the fifth rib was shingled to provide a larger window. The tumor was then shelled out from its pseudo capsule in its entirety (Figure 5). The patient tolerated the procedure well and was discharged with no complications.

Pathology report

**Figure 6:**
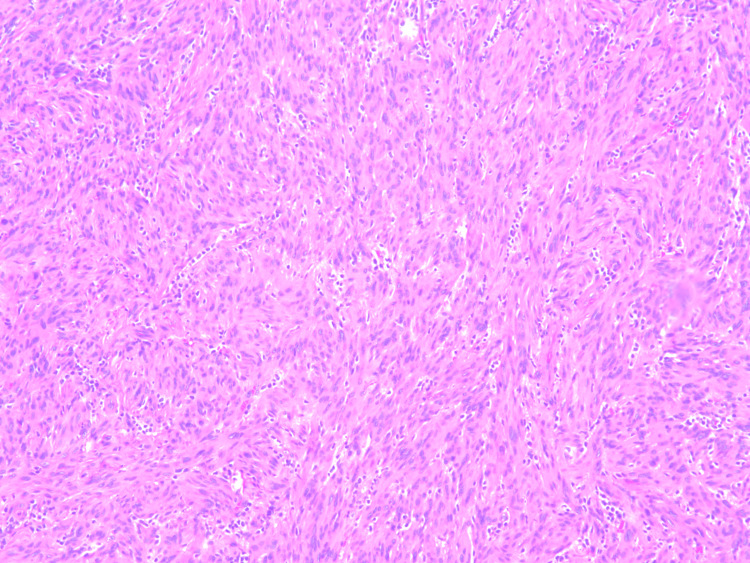
H&E stain of the sectioned schwannoma H&E: Hematoxylin and eosin

The pathology report revealed a schwannoma of spindle cell origin (Figure 6). The tumor measured 6.0 x 3.0 x 2.5 cm. It was described as a partially encapsulated mass that was pedunculated with a predominantly smooth, intact yellow-tan capsular surface. The cut surfaces are variegated with white, tan, and yellow colors. The mass was hemorrhagic, and the tissue texture ranged from soft and fleshy to firm. 

**Figure 7:**
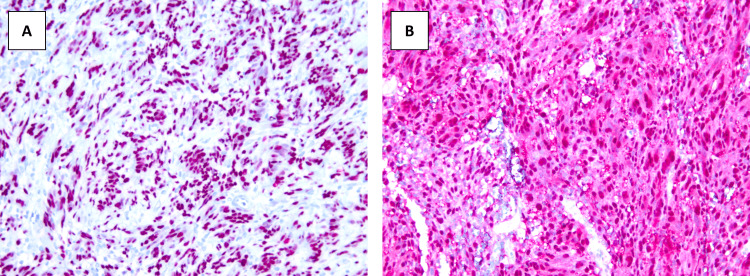
Immunohistochemical stains of the sectioned schwannoma A: SOX10; B: S100

Immunohistochemistry analysis revealed diffusely immunoreactive cells with SOX10 and S100 markers, supporting a repeat schwannoma diagnosis (Figures 7A, 7B).

**Figure 8:**
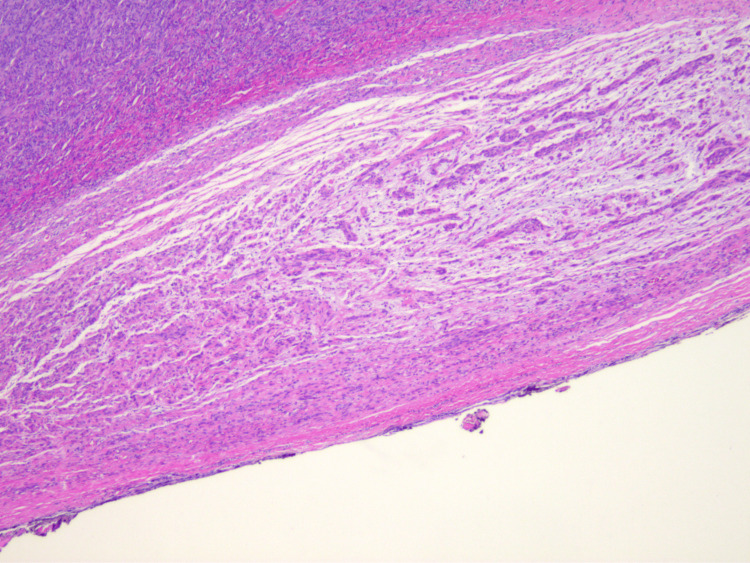
Schwannoma adjacent to a peripheral nerve

In some sections, parts of the schwannoma were identified as adjacent and associated with peripheral neurons (Figure 8).

## Discussion

A schwannoma is a slow-growing tumor formed from Schwann cells that myelinate the peripheral nervous system [6]. Due to variability in presentation, schwannoma subtypes are distinguished according to their location and microscopic features. Schwannomas often appear as asymptomatic, incidental findings on chest imaging, and also have been associated with mutations in the tumor suppressor gene NF2 [6]. Although schwannomas can be diagnosed at any age, most cases “have a peak incidence in the fourth to sixth decades of life” [6].

An intercostal schwannoma can be identified using a CT scan or magnetic resonance imaging (MRI). It grossly appears as a firm, encapsulated mass within the intercostal nerve of the thoracic trunk. If a biopsy of the sample tissue is collected, an intercostal schwannoma conventionally appears as compact spindle cells in palisades (Antoni A areas) admixed with hypocellular myxoid Antoni B areas [7]. These cells will show positive immunohistochemical staining for S-100 and SOX10. Like intercostal schwannomas, plexiform schwannomas are associated with NF2 and exhibit the immunohistochemical marker S-100. However, this variant typically lacks a “well-formed capsule and thick-walled vessels” and has a preference for the superficial soft tissue in the head and neck region [6]. Ultimately, increased cellularity, a multinodular or plexiform growth pattern, and a lack of a defined capsule are features that set a plexiform schwannoma apart from other schwannoma variants [8]. 

As with many other tumors of the chest wall, an intercostal schwannoma is treated by surgical resection. Alternative treatment approaches include observation, radiation therapy, and the use of antiangiogenics [7]. Recurrence of intercostal schwannomas following surgery is generally considered rare. Subtotal tumor resection, malignant histopathology, neurofibromatosis type II, large tumor size, and tumor configuration (e.g., dumbbell vs. non-dumbbell) have all been identified as risk factors for recurrence [5,9]. In the case of our patient, limited access to previous medical records leaves much room for speculation. That being said, the aforementioned risk factors potentially could have played a role in the uniqueness of this case. Nonetheless, limited literature currently exists on recurrent intercostal schwannomas and the associated biomarkers. This case presents an opportunity to reconsider how peripheral nerve schwannomas present and how they can be followed.

## Conclusions

Schwannomas are typically solitary and are characteristically found in the head, neck, mediastinum, and extremities. Complete surgical excision is the mainstay of treatment. However, we present an unusual case of recurring intrathoracic schwannoma originating from intercostal nerves, taking place over an extended period of 11 years. 

Although schwannomas usually have a low recurrence rate following complete surgical resection, this patient’s presentation demonstrates that reappearances can happen and that physicians must remain vigilant for new masses. Given the risk of delayed diagnosis due to the tumor's location and its slow-growing nature, this case advocates for the importance of long-term follow-up through regular clinical evaluations. Furthermore, there should be a lower threshold for repeat imaging in patients presenting with an intrathoracic mass who have previously undergone resection of a schwannoma. With only a limited number of cases reported of this occurrence, additional management guidelines should be considered to identify and treat patients at higher risk for recurrence.
